# Nanoantidote for repression of acidosis pH promoting COVID‐19 infection

**DOI:** 10.1002/VIW.20220004

**Published:** 2022-05-01

**Authors:** Qidong Liu, Huitong Ruan, Zhihao Sheng, Xiaoru Sun, Siguang Li, Wenguo Cui, Cheng Li

**Affiliations:** ^1^ Department of Anesthesiology and Perioperative Medicine Shanghai Fourth People's Hospital, School of Medicine, Tongji University Shanghai P. R. China; ^2^ Key Laboratory of Spine and Spinal Cord Injury Repair and Regeneration of Ministry of Education Orthopedic Department, Tongji Hospital, School of Medicine, Tongji University Shanghai P. R. China; ^3^ Department of Orthopaedics Shanghai Key Laboratory for Prevention and Treatment of Bone and Joint Diseases Shanghai Institute of Traumatology and Orthopaedics Ruijin Hospital Shanghai Jiao Tong University School of Medicine Shanghai P. R. China; ^4^ Department of Anesthesiology Shanghai First Maternity and Infant Hospital, School of Medicine, Tongji University Shanghai P. R. China

**Keywords:** ACE2, acidosis, antacid, COVID‐19, F‐actin, nanoantidote, SARS‐CoV‐2

## Abstract

Acidosis, such as respiratory acidosis and metabolic acidosis, can be induced by coronavirus disease 2019 (COVID‐19) infection and is associated with increased mortality in critically ill COVID‐19 patients. It remains unclear whether acidosis further promotes SARS‐CoV‐2 infection in patients, making virus removal difficult. For antacid therapy, sodium bicarbonate poses great risks caused by sodium overload, bicarbonate side effects, and hypocalcemia. Therefore, new antacid antidote is urgently needed. Our study showed that an acidosis‐related pH of 6.8 increases SARS‐CoV‐2 receptor angiotensin‐converting enzyme 2 (ACE2) expression on the cell membrane by regulating intracellular microfilament polymerization, promoting SARS‐CoV‐2 pseudovirus infection. Based on this, we synthesized polyglutamic acid‐PEG materials, used complexation of calcium ions and carboxyl groups to form the core, and adopted biomineralization methods to form a calcium carbonate nanoparticles (CaCO_3_‐NPs) nanoantidote to neutralize excess hydrogen ions (H^+^), and restored the pH from 6.8 to approximately 7.4 (normal blood pH). CaCO_3_‐NPs effectively prevented the heightened SARS‐CoV‐2 infection efficiency due to pH 6.8. Our study reveals that acidosis‐related pH promotes SARS‐CoV‐2 infection, which suggests the existence of a positive feedback loop in which SARS‐CoV‐2 infection‐induced acidosis enhances SARS‐CoV‐2 infection. Therefore, antacid therapy for acidosis COVID‐19 patients is necessary. CaCO_3_‐NPs may become an effective antacid nanoantidote superior to sodium bicarbonate.

## Graphical abstract

Acidosis‐related pH promotes SARS‐CoV‐2 pseudovirus infection by increasing cell membrane ACE2 expression. A CaCO_3_‐NPs antacid nanoantidote restored the normal blood related pH 7.4, attenuating the SARS‐CoV‐2 pseudovirus infection. This study suggests the existence of a positive feedback loop in which SARS‐CoV‐2 infection‐induced acidosis enhances further SARS‐CoV‐2 infection. CaCO_3_‐NPs nanoantidote may become an effective antacid.

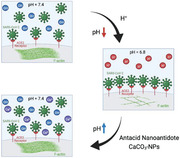

## 1 INTRODUCTION

COVID‐19 is caused by SARS‐CoV‐2, a novel coronavirus that leads to many symptoms, from asymptomatic to critical illness and even death.^1^ COVID‐19 patients who die are often critically ill.^2^ In severe cases, the disease can lead to acute respiratory distress syndrome, metabolic acidosis, and multiple organ dysfunction syndromes, among other complications.^3^ Metabolic acidosis, especially diabetic ketoacidosis (DKA), in COVID‐19 patients has been widely concerning.^4^ SARS‐CoV‐2 infection not only induces DKA in those with diabetes but also directly causes diabetes mellitus and DKA.^2^, ^4^, ^5^ Additionally, COVID‐19 infection also induces respiratory acidosis in critically ill patients.^6^ To rapidly reverse acute acidemia, the mainstay of therapy in the past placed great emphasis on intravenous sodium bicarbonate.^7^ Sodium bicarbonate decreases the primary composite outcome and day 28 mortality in an a priori‐defined stratum of patients with acute kidney injury.^8^ However, the use of sodium bicarbonate infusion is also controversial. More data argue against the bicarbonate. The adverse effect of sodium bicarbonate is considered to be the administered sodium load, which can induce hypervolemia, hyperosmolarity, and hypernatremia.^9^ Bicarbonate is also associated with delayed ketone clearance and worsened hypokalemia.^7^ Metabolic alkalosis, hypernatremia, and hypocalcemia are observed more frequently in bicarbonate‐treated patients.^8^, ^10^ Hypocalcemia may also lead to cardiovascular disease.^11^ Previous study proposed a Mg‐based micromotor for regulating the pH in stomach.^12^ However, further research is needed to develop new and safer antacid antidote drugs to balance the blood pH of patients with acidosis, especially acidosis induced by COVID‐19.

Although it is known to cause serious complications, it is unclear whether acidosis promotes SARS‐CoV‐2 virus infection, causing a vicious cycle and worsening COVID‐19. For SARS‐CoV‐2 virus infection, the first step is binding of the viral trimeric spike protein to the membrane‐bound form of the cellular receptor angiotensin‐converting enzyme 2 (ACE2).^13^ The ACE2 recombinant protein shows a great potential for inhibiting SARS‐CoV‐2 infection.^14^ Our previous study developed an inhaled ACE2‐engineered microfluidic microsphere for neutralization of COVID‐19.^15^ ACE2 is expressed not only in lung, but also in blood vessels, as well as in other tissues.^15^, ^16^ Especially, the lung has many vascular networks for pulmonary circulation.^17^ There is vasculopathy and vascular thrombosis in lungs from both humans and rhesus macaques infected with SARS‐CoV‐2.^18^ SARS‐CoV‐2 can directly infect human blood vessel cells.^19^ SARS‐CoV‐2 infection causes vascular endothelial cell dysfunction and vascular endothelial injury.^18^, ^20^ Higher cellular ACE2 expression on the membrane is more conducive to virus infection. SARS‐CoV‐2 utilizes some means to enhance infection efficiency. A previous study indicated that SARS‐CoV‐2 exploits species‐specific interferon‐driven upregulation of ACE2 to enhance virus infection.^21^ ACE2 can be significantly upregulated after SARS‐CoV‐2 infection by stimulation with inflammatory cytokines.^22^ These studies suggested that after infection, COVID‐19 may induce upregulation of ACE2 in cells to promote further infection for SARS‐CoV‐2 virus. In arterial vessels, a decrease in arterial blood pH directly indicted acidosis.^23^ However, it remains largely unknown whether the decrease in blood pH in patients with COVID‐19‐induced acidosis promotes SARS‐CoV‐2 infection by regulating expression of ACE2 on the cellular membrane, causing deterioration in critically ill patients.

ACE2 exists in two forms: cell membrane and plasma (soluble).^24^ Membrane ACE2 levels can be decreased by angiotensin II, which induces ACE2 transport to the perinuclear space.^25^ Rearrangement of the actin cytoskeleton has been shown to play important roles in intracellular trafficking.^26^ ACE2 is reported to localize with actin. The actin bundling protein fascin‐1 regulates the expression levels and subcellular localization of ACE2.^27^ With regard to infection by another kind of human coronavirus, NL63, which also needs to interact with ACE2, actin cortex remodeling is required for virus endocytosis.^28^ Changes in pH can influence actin polymerization and depolymerization.^29^ Indeed, interaction of the actin‐depolymerizing factor (ADF)/cofilin family and F‐actin is markedly pH dependent.^30^ Arterial blood pH ranges between 7.35 and 7.45 under normal physiological conditions,^31^ whereas that of acidosis patients is lower than 7.35.^23^ In some cases, it decreases to 7.2 and even 6.8 in patients with acidosis induced by COVID‐19.^32^ Whether this apparent change in arterial pH value regulates the level of membrane ACE2 is unknown.

In this study, we first discovered that acidosis‐related pH apparently promotes the infection efficiency of SARS‐CoV‐2 pseudovirus to ACE2 overexpression HEK293T (HEK293T‐ACE2) cell, or human umbilical vein endothelial cell (HUVEC). According to this finding, we focused on the CaCO_3_ to explore the potential possibility of neutralizing acids. Recent years, materials science has shown its more and more advantages in the understanding, diagnosis, or treatment of the viral diseases.^33^ Some carefully designed nanomaterials have the potential direct function of fighting viruses.^34^ Based on our previous study experience about biomaterials,^35^ we synthesized polyglutamic acid‐polyethylene glycol (PGlu‐PEG) materials in which polyglutamate (Glu) provides carboxyl groups to interact with Ca^2+^, preventing the mineralization of large CaCO_3_ blocks, and PEG shell acts to prevent agglomeration and aggregation of particles. Then use complexation of calcium ions and carboxyl groups to form the core and adopted biomineralization methods to form a calcium carbonate nanoparticles CaCO_3_‐NPs. We found that this (CaCO_3_‐NPs) nanoantidote apparently prevents acidosis to reduce the SARS‐CoV‐2 infection efficiency enhanced by acidosis. We further studied the molecular mechanism and found that acidosis‐related pH promotes SARS‐CoV‐2 infection by increasing ACE2 expression on the membrane via inhibition of actin polymerization. Using the CaCO_3_‐NPs antidote suppressed the effects of acidosis‐related pH in promoting SARS‐CoV‐2 pseudovirus infection. Our research suggests that a “positive feedback loop,” that is, acidosis caused by SARS‐CoV‐2 infection may promote SARS‐CoV‐2 infection in patients, resulting in a vicious cycle. Therefore, it is important to perform antacid treatment for critically ill COVID‐19 patients. Given the existing problems of sodium bicarbonate, we propose that this CaCO_3_‐NPs is a new and better antidote for treatment of acidosis that inhibits infection of SARS‐CoV‐2.

## 2 RESULTS

### 2.1 Acidosis‐related acidic pH conditions induced higher SARS‐CoV‐2 viral infection efficiency than normal physiological pH conditions

**FIGURE 1:**
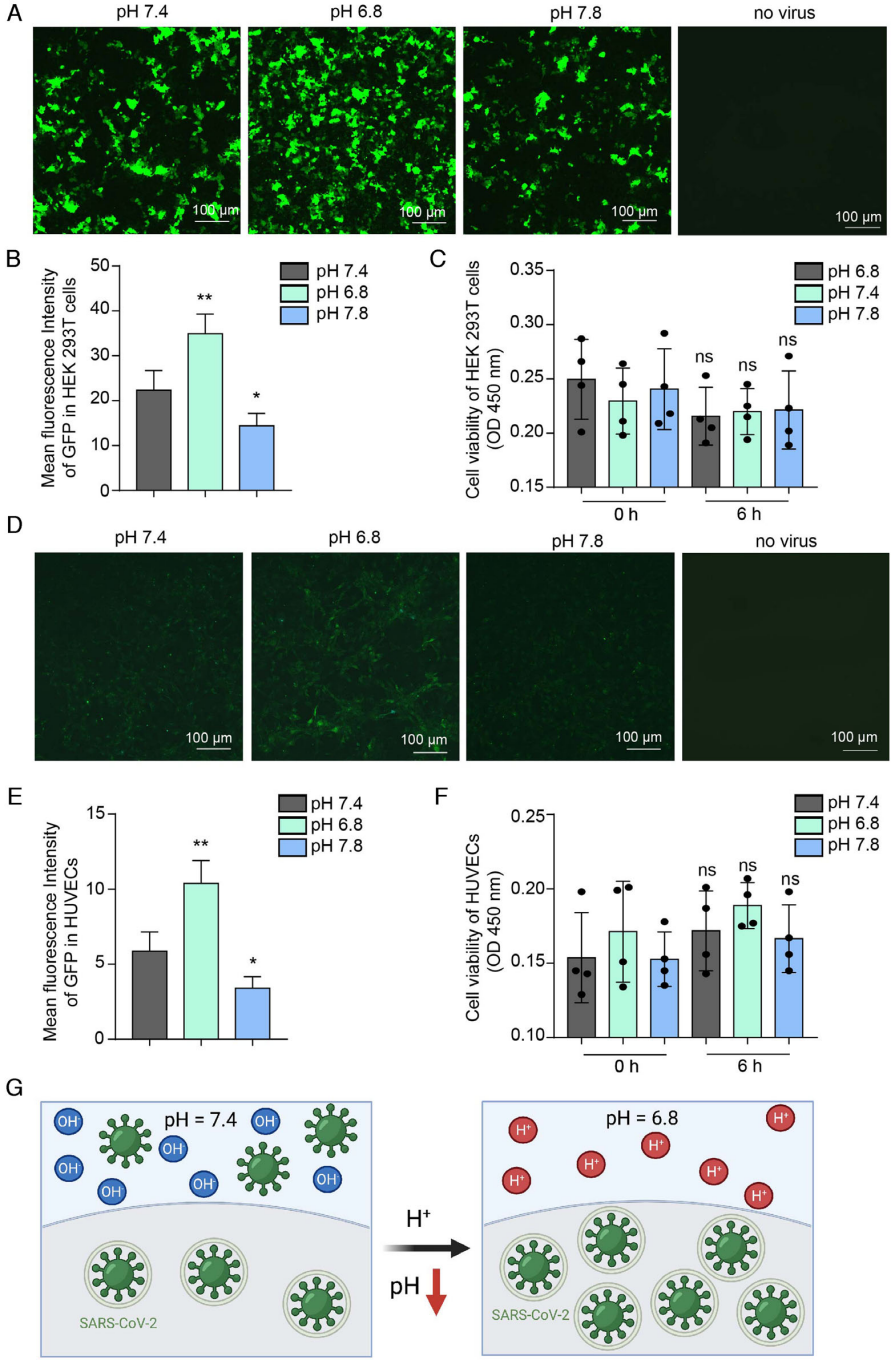
Acidic conditions result in a higher viral infection rate than alkaline conditions. (A) GFP fluorescence indicates SARS‐CoV‐2 pseudovirus infection in HEK293T‐ACE2 cells. The infection rate was higher at pH 6.8 than at pH 7.4 or 7.8. The group with no virus was used as the negative control, with no significant fluorescence of HEK293T‐ACE2 cells. (B) Statistics of the median fluorescence intensity of GFP in (A). (C) CCK‐8 assays indicated that different pH values (6.8, 7.4, and 7.8) did not influence cell viability during a 6‐h treatment. (D) SARS‐CoV‐2 pseudovirus infection of HUVECs in medium with different pH values. (E) Statistics of the median fluorescence intensity of GFP in (D). (F) CCK‐8 assays detected the viability of HUVECs at different pH values (6.8, 7.4, and 7.8) during a 6‐h treatment. The data shown are means ± SDs (*n* = 4). (G) Proposed consequences of acidic conditions: pH causes a higher viral infection rate than normal blood pH conditions. *p < .05, **p < .01. Asterisks (*) indicate statistical significance for the pH 6.8 and 7.8 groups compared with the pH 7.4 group; ns: no significant difference. Scale bar: 100 μm

We cultured human ACE2‐overexpressing HEK293T (HEK293T‐ACE2) cells with normal culture medium DMEM containing 10% fetal bovine serum (FBS). HEK293T‐ACE2 cell line can simulate the human cell expressing ACE2 protein for studying the SARS‐CoV‐2 virus infection. SARS‐CoV‐2 pseudovirus used its trimeric spike protein to bind with the ACE2 protein on HEK293T cell for further infection. HEK293T cell line can be continuously propagated for many passages, which facilitate the enough supplement of cells for the study. The next day after the cell seeding, we changed the culture medium to one at pH 6.8, pH 7.4, or pH 7.8 HEPES solution medium. Then added to a SARS‐CoV‐2 pseudovirus that has S protein and express the green fluorescent protein (GFP). Six hours later, we changed the culture medium to be normal fresh culture medium (DMEM, 10% FBS) for 48‐hour culture. GFP fluorescence was determined to indicate SARS‐CoV‐2 pseudovirus infection. We found that compared to the normal human blood‐related pH value of pH 7.4, the COVID‐19 patient acidosis‐related pH value (pH 6.8^32^) apparently increased the SARS‐CoV‐2 infection efficiency, as indicated by more GFP fluorescence (Figure 1A,B). We further found that pH 7.8 resulted in reduced efficiency compared to pH 7.4, which indicated weakly alkaline inhibition of virus infection (Figure 1A,B). To determine whether 6 h of treatment with medium at different pH values affects HEK293T‐ACE2 cell viability to influence virus infection, we performed a CCK‐8 assay and found no significant difference in cell viability after 6 h of treatment in different pH values (Figure 1C). ACE2 is expressed in the cardiovascular system.^16^ SARS‐CoV‐2 virus infects human blood vessel cells and causes vascular endothelial cell dysfunction and vascular endothelial injury.^18^, ^20^ Here, we investigated that whether pH value influences the SARS‐CoV‐2 virus infection of human blood vessel cells. So, we used the HUVECs, which express the ACE2 itself,^36^ and cultured in different pH value culture mediums to simulate the vascular endothelial cell in different pH value environments in blood vessel. We infected HUVECs with the SARS‐CoV‐2 pseudovirus and confirmed that acidic conditions promote infection (Figure 1D,E). Six hours of treatment with medium at different pH values also did not affect the viability of HUVECs (Figure 1F). These data indicate that acidosis‐associated acidic pH conditions significantly promote SARS‐CoV‐2 infection compared with the normal blood pH, which suggests a potential vicious cycle in patients with COVID‐19 acidosis (Figure 1G).

### 2.2 Schematic and characterization of antacid CaCO_3_‐NPs

**FIGURE 2:**
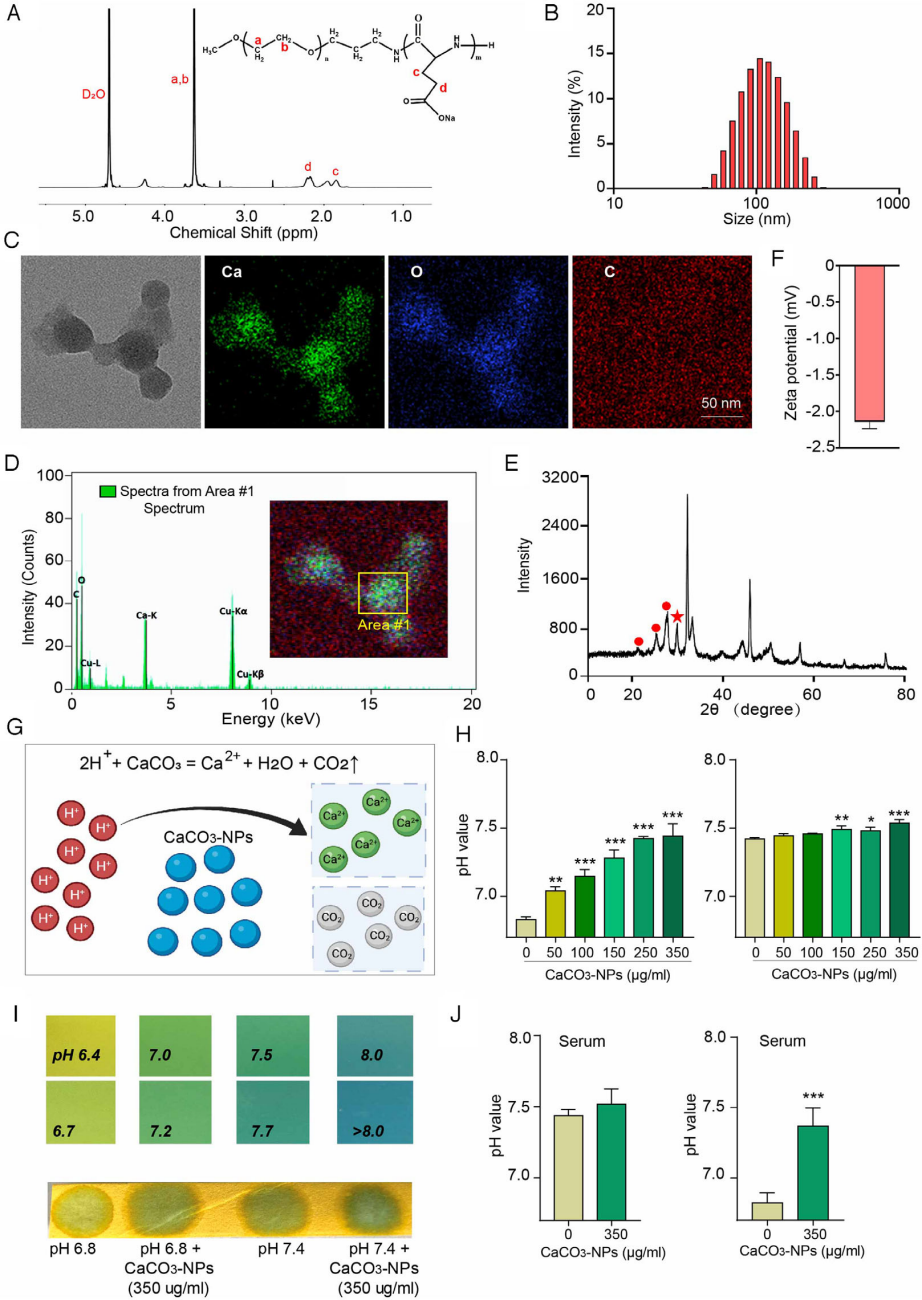
Schematic and characterization of antacid CaCO_3_‐NPs. (A) Characterization of mPEG‐P(Glu) copolymers by ^1^H‐NMR (300 MHz, in D_2_O). PEG (‐OCH_2_CH_2_, *δ* = 3.65 ppm), PGlu (‐CH_2_CH_2_COO‐: *δ* = 2.16 ppm, *δ* = 1.90 ppm). (B) Size distribution of CaCO_3_‐NPs measured by dynamic light scattering (DLS). (C) Morphology observation by TEM‐EDS of the CaCO_3_‐NPs. The scale bar represents 50 nm. (D) Elemental analysis of CaCO_3_‐NPs by TEM‐EDS in selected Area #1 (yellow box on the merged picture). (E) XRD curves of CaCO_3_‐NPs. Circle icon: vaterite, No. 33‐0268; pentagram icon: calcite, No. 05‐0586. (F) Detection of zeta potential of CaCO_3_‐NPs. (G) Schematics indicating the chemical reaction between CaCO_3_‐NPs and hydrogen ions showing how the nanoantidote plays a role in alleviating acidosis. (H) Different amounts of CaCO_3_‐NPs regulate the pH value of pH 6.8 (left panel) and pH 7.4 (right panel) solutions. (I) Precise pH test paper was used to assess CaCO_3_‐NPs (350 μg/ml final concentration) regulation of the pH value of pH 6.8 and pH 7.4 solutions. The upper image is a color chart of the pH value, and the bottom image is the color of the different experimental groups. (J) pH value of FBS was regulated by CaCO_3_‐NPs (350 μg/ml final concentration). Left image indicated the pH value of normal serum and normal serum with CaCO_3_‐NPs. Right image indicated the pH value of pH 6.8 serum and pH 6.8 serum with CaCO_3_‐NPs. The data shown are means ± SD (*n* = 4). *p < .05, **p <.01,***p < .001

To construct the antiacid nanodote, the functional polymer mPEG‐P(Glu) was synthesized, and the degree of polymerization was calculated to be 40 using ^1^H‐NMR. Characterization of mPEG‐P(Glu) copolymers: 300 MHz, in D_2_O, PEG: ‐OCH_2_CH_2_, *δ* = 3.65 ppm, PGlu: ‐CH_2_CH_2_COO‐: *δ* = 2.16 ppm, *δ* = 1.90 ppm (Figure 2A). Then, CaCO_3_‐NPs were prepared using mPEG‐P(Glu) block copolymers to interact with Ca^2+^ and CO_3_^2−^ in aqueous solution. Glu provides carboxyl groups to interact with Ca^2+^, preventing the mineralization of large CaCO_3_ blocks, and the PEG shell acts to prevent agglomeration and aggregation.^37^ The average diameter of the CaCO_3_‐NPs was approximately 100 nm, with a PDI value less than 0.2, indicating that the particles are uniform (Figure 2B). The morphology and element distribution of CaCO_3_‐NPs were analyzed by energy dispersive spectrometry (TEM‐EDS), showing that this nanoparticle was composed of Ca, C, and O and was homogeneous (Figure 2C,D). The X‐ray diffraction (XRD) analysis was also performed to investigate the crystalline structures of CaCO_3_‐NPs, and the result turned out to be vaterite and calcite (Figure 2E). We detected that the zeta potential (ZP) of CaCO_3_‐NP was −2.14 ± 0.09 mV. This weak negative charge of NPs suggested that there may be no significant interaction with proteins by interaction of charges and there may be low cytotoxicity (Figure 2F). CaCO_3_‐NPs were able to react with hydrogen ions (H^+^) to exert the acidic microenvironment (Figure 2G). We applied different amounts of CaCO_3_‐NPs (0, 50, 100, 150, 250, 350 μg/ml final concentration) to regulate the pH value of the pH 6.8 and pH 7.4 solutions. With increasing amounts of CaCO_3_‐NPs, the pH value of the pH 6.8 solution increased gradually and reached approximately pH 7.4 at 350 μg/ml. While for the pH 7.4 solution, the CaCO_3_‐NPs caused no significant changes in pH value (Figure 2H). Precise pH test paper was also used to assess how the CaCO_3_‐NPs (350 μg/ml) altered the pH value of the pH 6.8 or 7.4 solution (Figure 2I). Further, we detected the CaCO_3_‐NPs function in different pH values FBS to simulate the CaCO_3_‐NPs working in the blood, and determined whether the CaCO_3_‐NPs can neutralize the hydrogen ions to restore normal serum pH. First, we determined that the pH value of FBS is about 7.4, which was also similar with the pH of normal blood in human beings. 350 μg/ml CaCO_3_‐NPs in serum had no apparent influence on the serum pH value. In order to simulate the acidosis blood, the pH of serum was downregulated to 6.8 by adding concentrated hydrochloric acid slowly. Then the CaCO_3_‐NPs was added at the concentration of 350 μg/ml. It is found that the pH was apparently restored by supplementation with CaCO_3_‐NPs (Figure 2J).

### 2.3 CaCO₃‐NPs neutralize acid to inhibit viral infection of SARS‐CoV‐2

**FIGURE 3:**
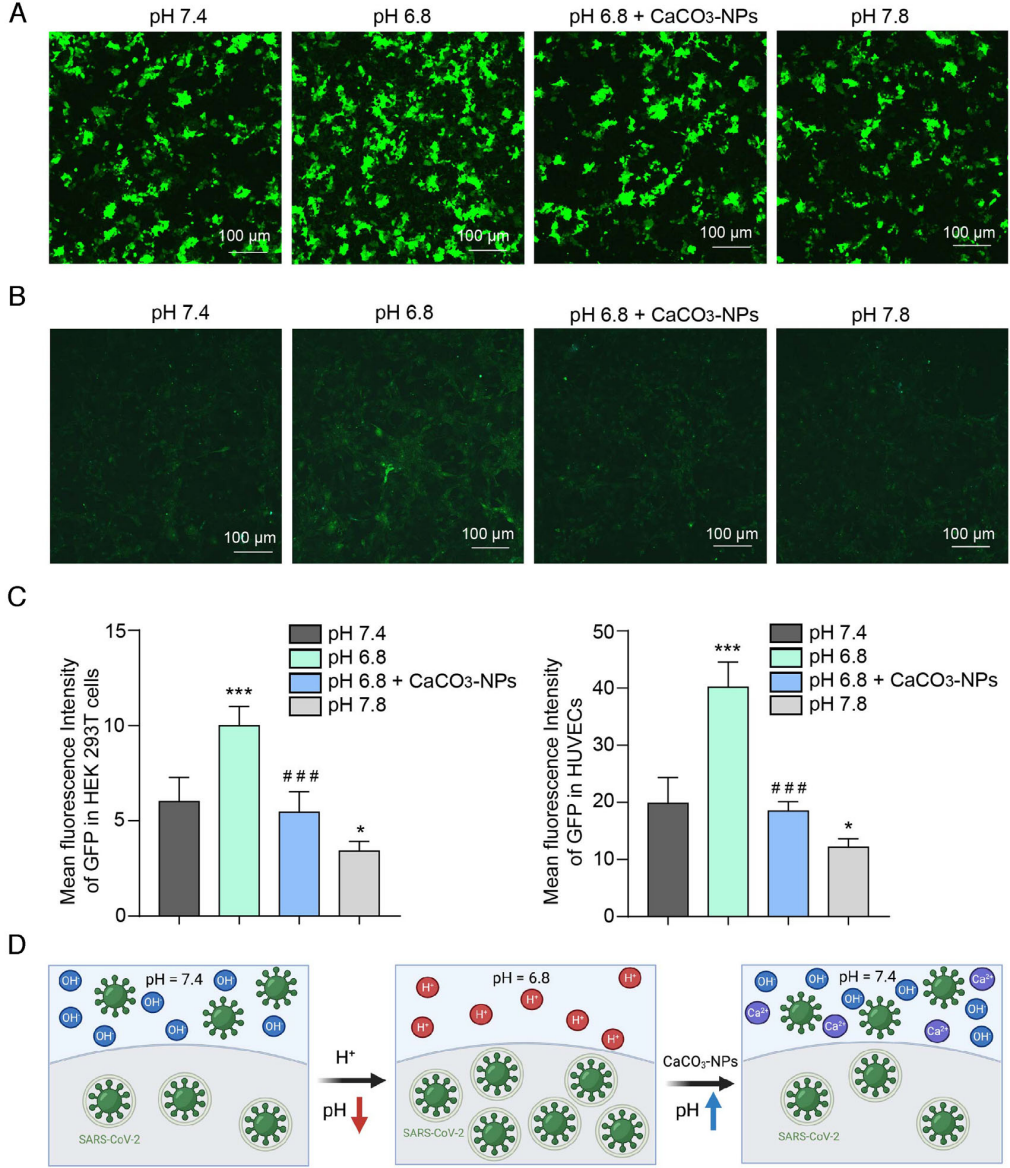
CaCO₃‐NPs‐mediated pH influences virus infection. (A) GFP fluorescence indicated that CaCO_3_‐NPs (350 μg/ml) attenuated the enhanced SARS‐CoV‐2 pseudovirus infection of HEK293T‐ACE2 cells and HUVECs caused by pH 6.8, as shown in (B). (C) Statistics of the median fluorescence intensity of GFP in HEK293T‐ACE2 cells (left panel) and HUVECs (right panel). (D) Proposed consequences of reduced viral infection rate due to CaCO_3_‐NPs neutralization of hydrogen ions under acidic conditions at the approximate blood pH (pH 7.4). The data shown are means ± SD (*n* = 4). * p<.05, *** or ^# # #^ indicates *p* < .001. Asterisks (*) indicate statistical significance for the pH 6.8 and 7.8 groups compared with the pH 7.4 group. Hashes (^#^) indicate statistical significance for the pH 6.8+CaCO₃‐NPs group compared with the pH 6.8 group. Scale bar: 100 μm

We added CaCO₃‐NPs (final concentration 350 μg/ml) to pH 6.8 medium and observed an apparent attenuation of the promotion of virus infection caused by pH 6.8. The infection efficiency was restored to approximately the level of that of the pH 7.4 medium. Similar in the HEK293T‐ACE2 cell line, CaCO₃‐NPs (350 μg/ml) inhibited virus infection efficiency in HUVECs at pH 6.8 (Figure 3A–C). These results not only confirm that the acidic condition promotes virus infection but also indicate the effectiveness of the CaCO₃‐NPs in attenuating virus infection (Figure 3D).

### 2.4 Acidic pH conditions induced more ACE2 expression on the cell membrane than in the cytoplasm

**FIGURE 4:**
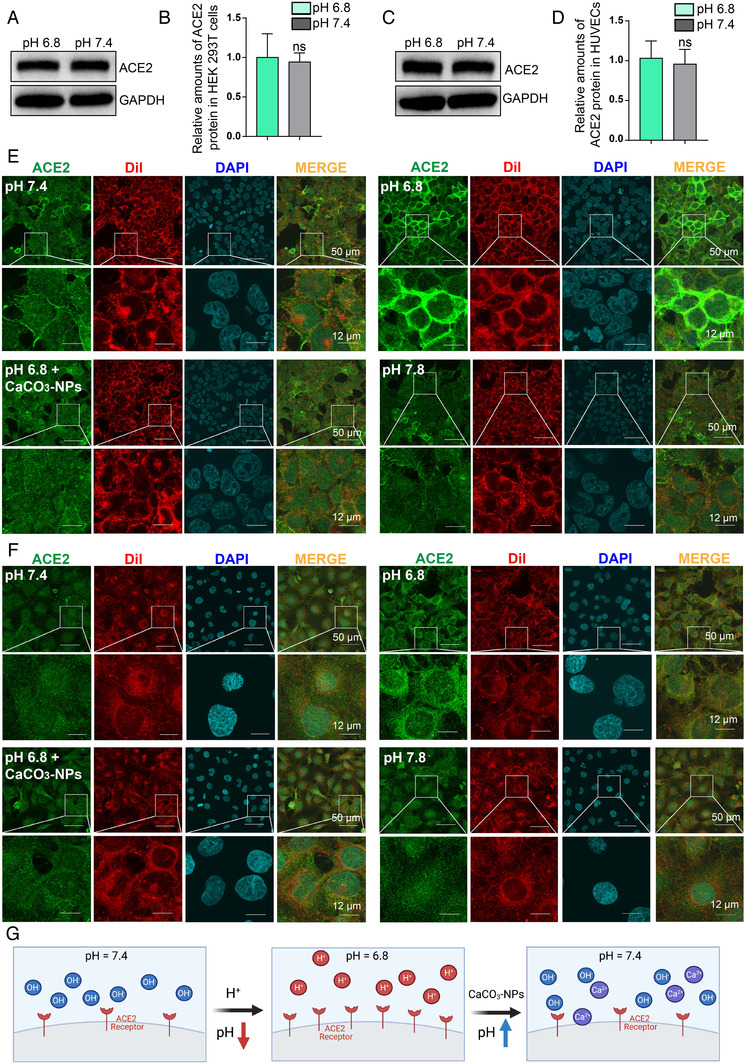
Acidic pH conditions induce a higher expression level of ACE2 on the cell membrane than in the cytoplasm. (A) Representative Western blots indicate that neither pH 6.8 nor pH 7.4 conditions changed the whole level of ACE2 expression in HEK293T‐ACE2 cells. (B) The statistics of the relative gray value ratio of ACE2 expression in (A). (C) Representative Western blots indicate that neither pH 6.8 nor pH 7.4 conditions changed the whole level of ACE2 expression in HUVECs. (D) The statistics of the relative gray value ratio of ACE2 expression in (C). (E) Immunofluorescence staining of ACE2 protein (green) indicated that compared with pH 7.4 or pH 7.8, pH 6.8 increased levels of ACE2 on the membrane of HEK293T‐ACE2 cells and (F) HUVECs, which was attenuated by CaCO_3_‐NPs (350 μg/ml). Dil staining showed the cell membrane (red), and DAPI staining showed the nucleus (blue). (G) Proposed consequences of CaCO_3_‐NPs neutralizing hydrogen ions under acidic conditions to counteract the effects of pH 6.8 increasing levels of ACE2 on the membrane. Scale bar: 50 μm (upper) or 12 μm (lower). The data shown are means ± SD (*n* = 4). ns: no significant difference of statistics

To determine whether the different infection efficiencies of SARS‐CoV‐2 under different pH conditions are caused by pH‐dependent ACE2 expression regulation in cells, we treated HEK293T‐ACE2 cells (Figure 4A,B) or HUVECs (Figure 4C,D) and found no apparent difference in total ACE2 protein expression under the different pH conditions. The mechanism for SARS‐CoV‐2 infection involves requisite binding of the virus to the membrane‐bound form of ACE2.^38^ Therefore, we further performed immunofluorescence staining to detect ACE2 and determined that the level of ACE2 on the cell membrane of HEK293T‐ACE2 cells (Figure 4E) and HUVECs (Figure 4F) was significantly increased under pH 6.8 conditions. In contrast, supplementation with CaCO₃‐NPs attenuated results with the pH 6.8 condition to a level similar to that of the pH 7.4 group (Figure 4E,F). These results show that ACE2 expression was induced by acidic‐related pH conditions and attenuated by CaCO₃‐NPs, which not only uncovers the mechanism by which acidic pH promotes SARS‐CoV‐2 infection but also suggests that CaCO₃‐NPs are a potential antidote to prevent SARS‐CoV‐2 infection in acidosis patients (Figure 4G).

### 2.5 Acidic pH conditions are not conducive to F‐actin polymerization

**FIGURE 5:**
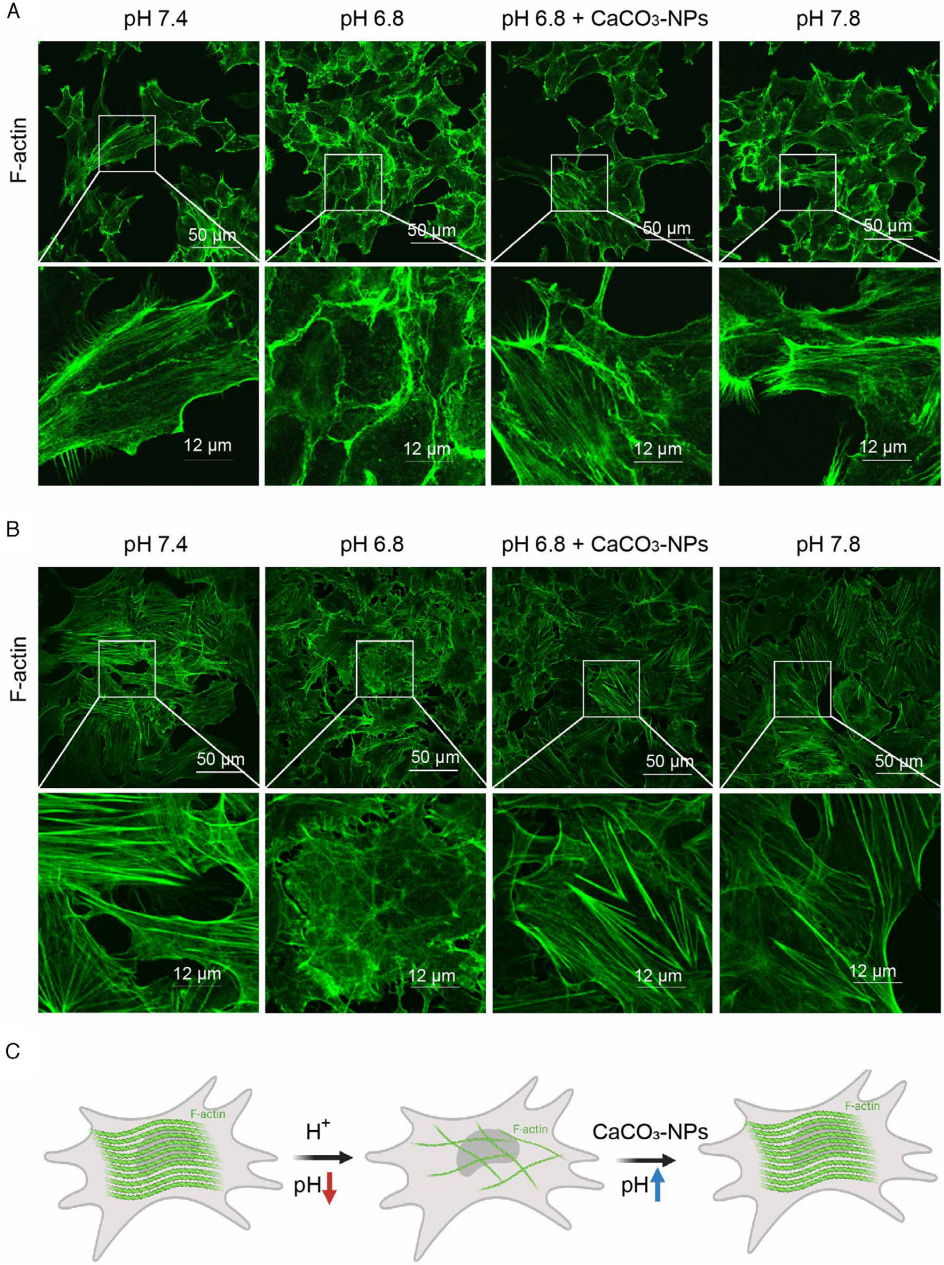
Acidic pH represses F‐actin polymerization, which is restored by CaCO₃‐NPs to normal blood pH conditions. (A) Immunofluorescence of F‐actin (phalloidin labeling) showed that there was more actin polymerization to form the large F‐actin bundle in HEK293T‐ACE2 cells and (B) HUVECs at pH 7.4, the normal blood pH condition, than in pH 6.8 medium. CaCO₃‐NPs restored the F‐actin bundle in pH 6.8 medium. (C) Proposed consequences of neutralization of hydrogen ions by CaCO_3_‐NPs to upregulate the pH value to restore the F‐actin polymerization repressed by pH 6.8. Scale bar: 50 μm (upper) or 12 μm (lower)

ACE2 is reported to colocalize with actin. The actin bundle protein fascin‐1 regulates the expression level and subcellular localization of ACE2.^27^ Thus, we detected the morphology of F‐actin by phalloidin labeling green fluorescence staining and found obvious actin polymerization and stable actin bundles in HEK293T‐ACE2 cells (Figure 5A) and HUVECs (Figure 5B) at pH 7.4, that is, normal blood pH conditions. Additionally, polymerization of F‐actin was stable in pH 7.8 medium. However, pH 6.8 induced a fuzzy dispersion morphology of F‐actin in cells (Figure 5A,B). Addition of CaCO₃‐NPs neutralized the hydrogen ions in the pH 6.8 medium and restored the apparent actin bundles in HEK293T‐ACE2 cells (Figure 5A) and HUVECs (Figure 5B), which was similar to the effects of pH 7.4 medium. This result reveals the effect of pH on regulating actin polymerization and stabilization (Figure 5C).

### 2.6 Promotion of actin polymerization inhibits ACE2 expression on the cell membrane and SARS‐CoV‐2 infection

**FIGURE 6:**
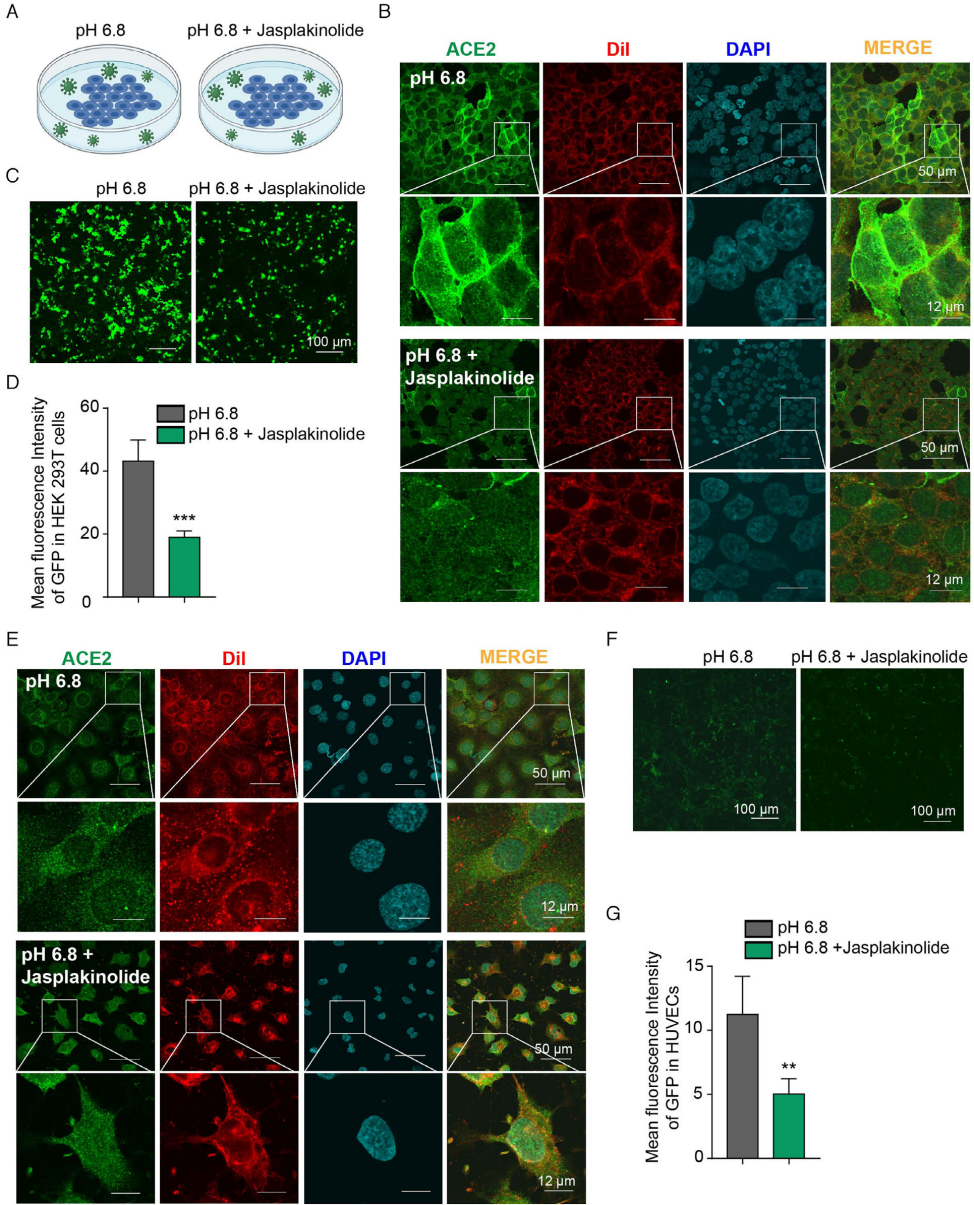
Actin polymerization reduces ACE2 levels on the cell membrane and SARS‐CoV‐2 pseudovirus infection. (A) Schematic illustration of the strategy by which SARS‐CoV‐2 infection is regulated by inducing F‐actin polymerization and stabilization with jasplakinolide in pH 6.8 medium. (B) Jasplakinolide (10 μM) reduces membrane ACE2 increased by pH 6.8 in HEK293T‐ACE2 cells. (C) GFP fluorescence indicates that jasplakinolide (10 μM) reverses the effects of the pH 6.8 condition that promotes pseudovirus infection in HEK293T‐ACE2 cells. (D) The statistics of the median fluorescence intensity of GFP in (C). (E) Jasplakinolide (10 μM) reduces HUVECs membrane ACE2 levels that are increased under pH 6.8 conditions. (F) GFP fluorescence indicates that jasplakinolide reverses the effects of the pH 6.8 condition that promotes pseudovirus infection in HUVECs. (G) The statistics of the median fluorescence intensity of GFP in (F). Scale bar: 50 μm (upper) or 12 μm (lower). The data shown are means ± SD (*n* = 4). **p < .01, ***p < .001

To assess whether induction of actin polymerization influences ACE2 expression on the cell membrane and even regulates SARS‐CoV‐2 infection, we added the jasplakinolide^39^ (10 μM), the widely used inducer of actin polymerization and F‐actin stabilization, to HEK293T‐ACE2 cells and HUVECs (Figure 6A). We observed an apparent reduction in ACE2 expression on the membrane in HEK293T‐ACE2 cells after jasplakinolide supplementation in pH 6.8 medium compared with cells cultured in pH 6.8 medium (Figure 6B). GFP fluorescence of pseudovirus infection indicated that jasplakinolide apparent attenuated SARS‐CoV‐2 pseudovirus infection efficiency enhanced by the pH 6.8 condition in HEK293T‐ACE2 cells (Figure 6C,D). In addition, jasplakinolide inhibited the increase in ACE2 expression on the HUVECs membrane (Figure 6E) and virus infection (Figure 6F,G) in pH 6.8 medium. These results indicate that SARS‐CoV‐2 infection is promoted by acidic conditions through decreased F‐actin polymerization, which is associated with the regulation of ACE2 expression on the cell membrane.

**FIGURE 7:**
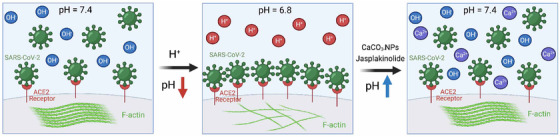
The proposed consequence of the promotion of SARS‐CoV‐2 infection by acidic pH, which is inhibited by CaCO₃‐NPs‐mediated pH increase or jasplakinolide. Acidosis‐related pH increases SARS‐CoV‐2 receptor ACE2 levels on the cell membrane, resulting in a significant increase in virus infection efficiency. Therefore, CaCO₃‐NPs can be used as an “antidote” of acidosis to neutralize hydrogen ions, effectively reversing and maintaining the acidic microenvironment to attenuate SARS‐CoV‐2 infection promoted by acidic conditions

The above results indicate that acidosis‐related pH can increase SARS‐CoV‐2 receptor ACE2 expression on the cell membrane, resulting in a significant increase in infection efficiency. Therefore, CaCO₃‐NPs can be used as an “antidote” for acidosis to neutralize hydrogen ions, effectively reversing and maintaining the acidic microenvironment to attenuate SARS‐CoV‐2 infection promoted by acidic conditions (Figure 7).

## 3 CONCLUSION

In this study, we found that acidosis‐related acidic pH benefits SARS‐CoV‐2 pseudovirus infection by increasing actin polymerization‐associated ACE2 expression on the membrane. Our results suggest the existence of a positive feedback loop in which SARS‐CoV‐2 infection‐induced acidosis enhances SARS‐CoV‐2 infection. We further report that CaCO_3_‐NP is a new potential antacid nanoantidote for acidosis in COVID‐19 patients.

SARS‐CoV‐2 infection induces metabolic acidosis^32^ and respiratory acidosis caused by acute respiratory distress syndrome.^9^, ^40^ Patients with DKA have an overall mortality rate of 37%, higher than that for all patients without DKA and patients with diabetes but without DKA.^41^ However, whether acidosis in critically ill COVID‐19 patients promotes further infection and difficult elimination of SARS‐CoV‐2 remains largely unknown. In this study, we mimicked the decrease in arterial blood pH in patients with acidosis to approximately pH 6.8 and found that compared with the normal physiological pH of 7.4, the virus infection rate approximately doubled under pH 6.8 conditions. These results confirm a positive feedback loop through which SARS‐CoV‐2 induces acidosis, further enhancing infection. Our results also suggest the necessity of antacid treatment for COVID‐19 patients with acidosis.

Increasing expression of the ACE2 protein promotes COVID‐19 infection.^42^ Downregulation of ACE2 decreases SARS‐CoV‐2 infection in vitro.^19^ ACE2 expression level on cell membrane is dynamically regulated.^25^ We found that a pH change (pH 7.4 or 6.8) did not significantly affect ACE2 total protein expression but that acidic pH increased ACE2 levels on the cell membrane. This not only explains why an increase in virus infection occurs under acidic conditions but also provides a simple and safe way to inhibit virus infection. Our study suggests that antacid therapy can reduce the increase in membrane ACE2 caused by acidosis‐related pH without changing expression of total ACE2 to reduce the efficiency of virus infection. In our study, regulation of pH with CaCO_3_‐NPs did not influence the total ACE2 protein level but only altered that on the cell membrane and in the cytoplasm. This maintains total ACE2 in cells to prevent side effects such as cardiovascular disease caused by ACE2 downregulation.

The actin bundling protein fascin‐1 regulates the expression levels and subcellular localization of ACE2.^27^ NL63, another kind of human coronavirus, infection also requires interaction with ACE2, and actin cortex remodeling is required for virus endocytosis.^28^ We found that a change in pH had a great influence on actin polymerization. At pH 7.4, thick and long F‐actin was observed in cells; at pH 6.8, actin was depolymerized, and a clear F‐actin structure could not be seen. Furthermore, the use of jasplakinolide, a classical molecule that promotes actin polymerization, significantly inhibited the increase in cell membrane ACE2 expression caused by pH 6.8 and the increase in SARS‐CoV‐2 pseudovirus infection efficiency. Our study suggests that depolymerization of actin under acidic conditions leads to an increase in ACE2 content in the cell membrane and promotes SARS‐CoV‐2 infection. The findings suggest the necessity of antacid therapy in COVID‐19 patients.

As an antacid, sodium bicarbonate has adverse effects, such as sodium load, which induces hypervolemia, hyperosmolarity, and hypernatremia,^9^ and bicarbonate is also associated with delayed ketone clearance and worsened hypokalemia.^7^ Hence, we used CaCO_3_ to neutralize hydrogen ions, which does not produce excessive sodium ions and bicarbonate. Additionally, hypocalcemia frequently occurs in COVID‐19 patients who have a worse outcome.^43^ Sodium overload caused by sodium bicarbonate aggravates hypocalcemia, which may lead to cardiovascular disease.^10^ Calcium supplement by oral or administration of CaCl_2_ is commonly used to treat hypocalcemia.^44^ Previous study indicated that the metal organic frameworks (MOFs) biomaterials release Ca^2+^ on demand to promote extracellular matrix mineralization and upregulate osteogenic gene expression.^45^ Therefore, in our study, a CaCO_3_‐NPs was used to neutralize hydrogen ions and as a calcium supplement to help alleviate hypocalcemia. Sodium bicarbonate is a weakly alkaline salt that is soluble in water and results in a weakly alkaline pH. In excess, the blood pH will rise, with possible alkalosis. However, CaCO_3_ does not significantly changes the blood acid–base balance under normal physiological conditions.^46^ So, compared with sodium bicarbonate, CaCO_3_ is relatively safe. Novel biomaterials treatment for COVID‐19 has the potential new strategies and opportunities.^47^ CaCO_3_‐NPs are rapidly degraded under a slightly acidic environment^48^ and have potential as drug delivery systems and may be administered through inhalation, intravenous injection, and topical application and so on,^49^ which suggests the CaCO_3_‐NPs being an efficient treatment strategy in the future. We utilized CaCO_3_‐NPs to neutralize the acidic environment under acidosis‐associated acidic pH conditions to repress SARS‐CoV‐2 virus infection. Compared with sodium bicarbonate, CaCO_3_‐NPs may be a better alternative for the treatment of acidosis in COVID‐19 patients.

Our work reveals that acidosis‐related acidic pH conditions promote SARS‐CoV‐2 infection. Mechanistically, a lower pH value increases ACE2 expression on the membrane by inhibiting actin polymerization. SARS‐CoV‐2 infection induces a positive feedback loop involving acidosis in COVID‐19 patients and increases ACE2 levels on the membrane to facilitate viral entry, which induces a vicious cycle in severely ill patients. Our study also suggests that it may be necessary to perform antacid therapy for acidosis in severely ill COVID‐19 patients. In view of the controversy over the use of sodium bicarbonate, we propose the potentially safer CaCO_3_‐NPs as treatment.

## 4 EXPERIMENTAL SECTION/METHODS

### 4.1 Cell culture

The HEK293T cell line with ACE2 overexpression (HEK293T‐ACE2 cells) (Genomeditech, GM‐C09233, China) was cultured in DMEM (Gibco) with 10% FBS (Lonsera, S711‐001S, Uruguay). HUVECs were cultured in RPMI‐1640 medium (Gibco) with 10% FBS. Cells were cultured at 37°C, 5% CO_2_ atmosphere.

### 4.2 Different pH value culture medium

The basic ingredients of the extracellular solution medium were as follows: 143 mM NaCl, 5 mM KCl, 10 mM HEPES, 10 mM glucose, 2 mM CaCl_2_, and 21 mM MgCl_2_. A NaOH solution was used to adjust the pH value of solutions, as monitored by using a pH meter (Mettler Toledo, FiveEasy Plus, Switzerland).

### 4.3 Infection of SARS‐CoV‐2 pseudovirus

SARS‐CoV‐2 spike pseudovirus (Genomeditech, batch number VF9436) based on the HIV lentivirus packaging system is a replication‐defective pseudovirus that contains the spike protein and expresses the GFP. Green fluorescence can be used to determine infection by the pseudovirus. The day before the experiment, HEK293T‐ACE2 cells were seeded into a 24‐well culture plate (1 × 10^5^ cells/well) for virus infection the next day. The pseudovirus was completely thawed at 4°C, and a 1:100‐fold dilution of the pseudovirus infection solution was used with different pH solutions. The diluted virus infection solution was added to the culture plate. After 6 h, the medium was replaced with normal DMEM (with 10% FBS) medium for culture for 48 h. Confocal microscopy (Olympus, FV3000, Japan) was used to observe GFP fluorescence.

### 4.4 Western blotting

Protein lysis buffer (M‐PER Mammalian Protein Extraction Reagent, Thermo, USA) was used to extract total cell protein. A protease inhibitor cocktail (Bimake, Shanghai, China) was added to the lysis buffer to prevent protein degradation. After the SDS‐PAGE electrophoresis of protein, the proteins were transferred onto polyvinylidene fluoride (PVDF) membranes (BioRad, USA) to detect the level of ACE2 expression. Anti‐ACE2 (21115‐1‐AP, 1:1000 dilution, Proteintech, USA) was used as the primary antibody, and anti‐GAPDH (ab8245, 1:1000 dilution, Abcam, USA) was used as a loading control. The secondary antibodies used were anti‐mouse IgG HRP‐linked antibody (7076 V, 1:3000 dilution; Cell Signaling Technology, USA) and anti‐rabbit IgG horseradish peroxidase (HRP)‐linked antibody (7074 V, 1:3000 dilution; Cell Signaling Technology). The ChemiDoc XRS+ system (Bio‐Rad) was applied to determine the protein level with enhanced chemiluminescence (Clarity Western ECL substrate, Bio‐Rad).

### 4.5 Immunofluorescence staining

HEK293T‐ACE2 cells or HUVECs were seeded onto polylysine (Beyotime, China)‐coated cover glass and fixed in 4% paraformaldehyde (PFA) solution for 18 min. Then, 0.2% Triton X‐100 solution was added for 8 min. The slides were blocked for 1 h in 10% FBS PBS solution. For detection of ACE2 expression, the cells were incubated with the anti‐ACE2 primary antibody (1:200 dilution, Proteintech), with rabbit IgG‐H&L (Alexa Fluor 594) (ab150080, 1:1000 dilution, Abcam) as the secondary antibody. Phalloidin Alexa Fluor 488 (A12379, Thermo, USA) was used to detect F‐actin. Dil (DIIC_18_(3), 1,1'‐dioctadecyl‐3,3,3',3'‐tetramethylindocarbocyanine perchlorate) (Yeasen, China) was used to stain the cell membrane (red), and the nucleus was stained (blue) with DAPI (Sigma, USA). Images were captured using a confocal microscope (Olympus).

### 4.6 Synthesis of methoxypoly(ethylene glycol)‐block‐poly(sodium glutamate) (mPEG‐P(Glu)) polymers

mPEG‐P(Glu) polymers were synthesized as previously described.^50^ Briefly, l‐glutamic acid γ‐benzyl ester (cat. 49510 Merck, USA) was reacted with triphosgene (cat. 330752, Merck, USA) to obtain the *N*‐carboxyanhydride of γ‐benzyl l‐glutamate (NCA‐BLG) via the Fuchs–Farthing method. Then, NCA‐BLG was polymerized in *N*,*N*‐dimethylformamide, as initiated by the amino group of CH_3_O‐PEG‐NH_2_ (Mw 12K, Xiamen Sinopeg Biotech Co., Ltd., China), to synthesize mPEG‐poly(**γ**‐benzyl l‐glutamate) (mPEG‐PBLG). Finally, the benzyl groups of mPEG‐PBLG were removed by mixing with 0.5 N NaOH at room temperature to obtain mPEG‐P(Glu) polymers, which were determined by ^1^H‐NMR spectroscopy (300 MHz; solvent: D_2_O).

### 4.7 Construction and characterization of CaCO_3_‐NPs

Nanoparticles were prepared via the biomineralization method.^51^ First, 0.5 ml mPEG‐P(Glu) (10 mg/ml) and 0.1 ml CaCl_2_ aqueous solution (100 mg/ml) were mixed. Then, 0.01 M Tris‐HCl buffer (pH 8.0) was slowly added to adjust the pH value to pH 7.8 to form Ca^2+^ chelate compounds, and 0.2 ml Na_2_CO_3_ solution (10 mg/ml) was added dropwise to the mixture until opalescence was observed, indicating the formation of CaCO_3_‐NPs. The mixture was stirred at 4°C overnight and centrifuged at 14,000 rpm for 15 min to remove excess ions and copolymers. The size distribution was characterized by DLS (Zetasizer Nano S, Malvern, UK), and the morphology and element compositions were observed by transmission electron microscopy (TEM, TALOS F200X), the XRD analysis was carried out by Aeris XRD instrument (Malvern Panalytical) and analyzed by MDI jade 6.0 software.

### 4.8 Statistical analysis

Student's *t*‐test was used when two independent groups were compared; one‐way ANOVA extended more than two groups. Detailed instructions are provided in figure legends, and *p*‐values <.05 were considered statistically significant. GraphPad Prism software (version 8.0; GraphPad Software, La Jolla, CA, USA) was used to calculate statistics.

## CONFLICT OF INTEREST

The authors declare that there is no conflict of interest.
